# Supplementary material for: Occurrence and characteristics of extended-spectrum-β-lactamase- and pAmpC-producing Klebsiella pneumoniae isolated from companion animals with urinary tract infections
Source: PLoS One. 2024 Jan 16;19(1):e0296709. doi: 10.1371/journal.pone.0296709 (PMC10790997; doi:10.1371/journal.pone.0296709)
Supplement: S1 Table — (DOCX) [file pone.0296709.s001.docx]

S1 Table. the allele number and sequence types (STs) of ESBL and/or pAmpC-producing *K. pneumoniae* analyzed by multilocus sequence typing (MLST)

| Isolate number | Species | Allele number | | | | | | | ST |
| --- | --- | --- | --- | --- | --- | --- | --- | --- | --- |
|  |  | *gapA* | *infB* | *mdh* | *pgi* | *phoE* | *rpoB* | *tonB* |  |
| 1337 | Cat | 1 | 1 | 1 | 1 | 1 | 1 | 4 | 709 |
| 1626 | Cat | 1 | 1 | 1 | 1 | 1 | 1 | 4 | 709 |
| 1917 | Cat | 3 | 1 | 1 | 1 | 1 | 1 | 1 | 265 |
| 1954 | Dog | 1 | 1 | 1 | 1 | 1 | 1 | 1 | 15 |
| 1994 | Dog | 2 | 1 | 19 | 4 | 9 | 4 | 34 | 967 |
| 2157 | Dog | 1 | 1 | 1 | 1 | 1 | 1 | 1 | 15 |
| 2165 | Dog | 2 | 9 | 2 | 1 | 13 | 1 | 16 | 37 |
| 2191 | Cat | 1 | 1 | 1 | 1 | 1 | 1 | 23 | 655 |
| 2233 | Cat | 2 | 1 | 97 | 1 | 9 | 4 | 13 | 846 |
| 2265 | Dog | 2 | 9 | 2 | 1 | 13 | 1 | 16 | 37 |
| 2277 | Dog | 3 | 4 | 6 | 1 | 7 | 4 | 4 | 273 |
| 2294 | Dog | 3 | 3 | 1 | 1 | 1 | 1 | 4 | 11 |
| 2517 | Dog | 2 | 3 | 6 | 1 | 9 | 4 | 13 | 592 |
| 2544 | Cat | 3 | 3 | 1 | 1 | 1 | 1 | 4 | 11 |
| 2551 | Dog | 2 | 3 | 2 | 2 | 6 | 4 | 4 | 29 |
| 2555 | Dog | 3 | 4 | 6 | 1 | 7 | 4 | 38 | 147 |
| 2561 | Cat | 3 | 3 | 1 | 1 | 1 | 1 | 4 | 11 |
| 2591 | Dog | 16 | 18 | 36 | 27 | 42 | 29 | 67 | unknown |
| 2648 | Dog | 2 | 1 | 2 | 1 | 1 | 1 | 68 | 966 |
| 2674 | Dog | 2 | 1 | 1 | 1 | 7 | 1 | 12 | 485 |
| 2697 | Dog | 2 | 1 | 1 | 1 | 7 | 1 | 12 | 485 |
| 2702 | Dog | 2 | 1 | 1 | 1 | 7 | 1 | 12 | 485 |
| 2715 | Cat | 2 | 1 | 2 | 1 | 10 | 1 | 4 | 469 |
| 2725 | Dog | 4 | 1 | 2 | 1 | 1 | 10 | 43 | 1995 |
| 2734 | Dog | 2 | 1 | 1 | 1 | 7 | 1 | 12 | 485 |
| 2750 | Dog | 3 | 3 | 1 | 1 | 1 | 1 | 4 | 11 |
| 2755 | Dog | 1 | 1 | 1 | 1 | 1 | 1 | 1 | 15 |
| 2768 | Dog | 2 | 1 | 1 | 1 | 12 | 27 | 1 | 1825 |
| 2777 | Dog | 2 | 1 | 1 | 1 | 12 | 27 | 1 | 1825 |
| 2812 | Dog | 2 | 5 | 2 | 2 | 1 | 4 | 753 | unknown |
| 2813 | Dog | 2 | 1 | 1 | 20 | 56 | 4 | 31 | 950 |
| 2814 | Dog | 2 | 1 | 1 | 1 | 12 | 27 | 1 | 1825 |
| 2815 | Cat | 2 | 1 | 2 | 1 | 1 | 4 | 123 | 2643 |
| 2830 | Dog | 1 | 1 | 1 | 1 | 1 | 1 | 1 | 15 |
| 2837 | Dog | 3 | 3 | 1 | 1 | 1 | 1 | 4 | 11 |
| 2844 | Dog | 2 | 1 | 1 | 1 | 7 | 1 | 12 | 485 |
| 2851 | Cat | 3 | 3 | 1 | 1 | 1 | 1 | 4 | 11 |
| 2855 | Dog | 3 | 4 | 6 | 1 | 7 | 4 | 38 | 147 |
| 2868 | Dog | 2 | 1 | 1 | 6 | 7 | 1 | 12 | 45 |
| 2872 | Cat | 1 | 1 | 1 | 1 | 1 | 1 | 23 | 655 |
| 2877 | Dog | 1 | 1 | 1 | 1 | 1 | 1 | 1 | 15 |
| 2880 | Dog | 2 | 9 | 2 | 1 | 13 | 1 | 16 | 37 |
| 2899 | Dog | 1 | 1 | 1 | 1 | 1 | 1 | 1 | 15 |
| 2900 | Dog | 3 | 3 | 1 | 1 | 1 | 1 | 4 | 11 |
| 2901 | Dog | 2 | 1 | 99 | 1 | 1 | 1 | 129 | 3393 |
| 2903 | Cat | 1 | 1 | 1 | 1 | 1 | 1 | 23 | 655 |
| 2904 | Cat | 1 | 1 | 1 | 1 | 1 | 1 | 23 | 655 |
| 2915 | Dog | 2 | 1 | 99 | 1 | 1 | 1 | 129 | 3393 |
| 2930 | Dog | 25 | 33 | 1 | 26 | 7 | 1 | 56 | 198 |
| 2936 | Dog | 2 | 5 | 1 | 1 | 9 | 1 | 212 | 1431 |
| 2937 | Cat | 1 | 1 | 1 | 1 | 1 | 1 | 23 | 655 |
| 2938 | Cat | 1 | 1 | 1 | 1 | 1 | 1 | 23 | 655 |
| 2947 | Dog | 18 | 22 | 56 | 61 | 11 | 13 | 51 | 3216 |
| 2953 | Dog | 2 | 1 | 99 | 1 | 1 | 1 | 129 | 3393 |
| 2956 | Dog | 1 | 1 | 114 | 166 | 1 | 146 | 1 | unknown |
| 2957 | Dog | 2 | 5 | 1 | 1 | 4 | 1 | 4 | 636 |
| 2962 | Cat | 10 | 1 | 1 | 1 | 9 | 4 | 112 | 2817 |
