# Supplementary material for: Occurrence and characteristics of extended-spectrum-β-lactamase- and pAmpC-producing Klebsiella pneumoniae isolated from companion animals with urinary tract infections
Source: PLoS One. 2024 Jan 16;19(1):e0296709. doi: 10.1371/journal.pone.0296709 (PMC10790997; doi:10.1371/journal.pone.0296709)
Supplement: S2 Table — (DOCX) [file pone.0296709.s002.docx]

S2 Table. Presence of the virulence genes of ESBL and/or pAmpC-producing *K*. *pneumoniae*

| Isolate number | Species | Virulence genes |
| --- | --- | --- |
| 1337 | Cat | *wabG, uge, entB, kfu, mrkD, fimH* |
| 1626 | Cat | *wabG, uge, entB, kfu, mrkD, fimH* |
| 1917 | Cat | *wabG, uge, entB, kfu, mrkD, fimH* |
| 1954 | Dog | *wabG, uge, entB, kfu, mrkD, fimH* |
| 1994 | Dog | *wabG, uge, entB, mrkD, fimH* |
| 2157 | Dog | *wabG, uge, entB, kfu, mrkD, fimH* |
| 2165 | Dog | *wabG, uge, entB, kfu, mrkD, fimH* |
| 2191 | Cat | *wabG, uge, entB, mrkD, fimH* |
| 2233 | Cat | *wabG, uge, entB, mrkD, fimH* |
| 2265 | Dog | *wabG, uge, entB, mrkD, fimH* |
| 2277 | Dog | *wabG, entB, mrkD, fimH* |
| 2294 | Dog | *wabG, uge, entB, mrkD, fimH* |
| 2517 | Dog | *rmpA, wabG, entB, iutA, mrkD, fimH* |
| 2544 | Cat | *wabG, uge, entB, mrkD, fimH* |
| 2551 | Dog | *wabG, uge, entB, mrkD, fimH* |
| 2555 | Dog | *wabG, uge, entB, mrkD, fimH* |
| 2561 | Cat | *wabG, uge, entB, mrkD, fimH* |
| 2591 | Dog | *wabG, uge, entB, kfu, mrkD, fimH* |
| 2648 | Dog | *wabG, uge, entB, mrkD, fimH* |
| 2674 | Dog | *wabG, uge, entB, mrkD, fimH* |
| 2697 | Dog | *wabG, uge, entB, mrkD, fimH* |
| 2702 | Dog | *wabG, uge, entB, mrkD, fimH* |
| 2715 | Cat | *wabG, uge, entB, mrkD, fimH* |
| 2725 | Dog | *wabG, uge, entB, iutA* |
| 2734 | Dog | *wabG, uge, entB, mrkD, fimH* |
| 2750 | Dog | *wabG, uge, entB, mrkD, fimH* |
| 2755 | Dog | *wabG, uge, entB, kfu, mrkD, fimH* |
| 2768 | Dog | *wabG, uge, entB, mrkD, fimH* |
| 2777 | Dog | *wabG, uge, entB, mrkD, fimH* |
| 2812 | Dog | *wabG, uge, entB, mrkD, fimH* |
| 2813 | Dog | *wabG, uge, entB, mrkD, fimH* |
| 2814 | Dog | *wabG, uge, entB, mrkD, fimH* |
| 2815 | Cat | *wabG, uge, entB, mrkD, fimH* |
| 2830 | Dog | *wabG, uge, entB, kfu, mrkD, fimH* |
| 2837 | Dog | *wabG, uge, entB, mrkD, fimH* |
| 2844 | Dog | *wabG, uge, entB, mrkD, fimH* |
| 2851 | Cat | *wabG, uge, entB, mrkD, fimH* |
| 2855 | Dog | *wabG, uge, entB, mrkD, fimH* |
| 2868 | Dog | *wabG, uge, entB, mrkD, fimH* |
| 2872 | Cat | *wabG, uge, entB, kfu, mrkD, fimH* |
| 2877 | Dog | *wabG, uge, entB, kfu, mrkD, fimH* |
| 2880 | Dog | *wabG, uge, entB, mrkD, fimH* |
| 2899 | Dog | *wabG, uge, entB, kfu, mrkD, fimH* |
| 2900 | Dog | *wabG, uge, entB, mrkD, fimH* |
| 2901 | Dog | *wabG, uge, entB, mrkD, fimH* |
| 2903 | Cat | *wabG, uge, entB, kfu, mrkD, fimH* |
| 2904 | Cat | *wabG, uge, entB, kfu, mrkD, fimH* |
| 2915 | Dog | *wabG, uge, entB, mrkD, fimH* |
| 2930 | Dog | *wabG, uge, entB, mrkD, fimH* |
| 2936 | Dog | *wabG, uge, entB, mrkD, fimH* |
| 2937 | Cat | *wabG, uge, entB, kfu, mrkD, fimH* |
| 2938 | Cat | *wabG, uge, entB, kfu, mrkD, fimH* |
| 2947 | Dog | *allS, wabG, uge, entB, kfu, mrkD, fimH* |
| 2953 | Dog | *wabG, uge, entB, mrkD, fimH* |
| 2956 | Dog | *wabG, uge, entB, kfu, mrkD, fimH* |
| 2957 | Dog | *wabG, uge, entB, mrkD, fimH* |
| 2962 | Cat | *wabG, uge, entB, kfu, mrkD, fimH* |
