# Supplementary material for: Occurrence and characteristics of extended-spectrum-β-lactamase- and pAmpC-producing Klebsiella pneumoniae isolated from companion animals with urinary tract infections
Source: PLoS One. 2024 Jan 16;19(1):e0296709. doi: 10.1371/journal.pone.0296709 (PMC10790997; doi:10.1371/journal.pone.0296709)
Supplement: S3 Table — (DOCX) [file pone.0296709.s003.docx]

S3 Table 3. Antimicrobial susceptibility testing of ESBL and/or pAmpC-producing *K. pneumoniae*

| Isolate  number | Species | Antimicrobials | | | | | | | | |
| --- | --- | --- | --- | --- | --- | --- | --- | --- | --- | --- |
|  |  | Amoxicillin | Ampicillin | Augmentin | Cefovecin | Cefixime | Cefpodoxime | Ceftazidime | Cefotaxime | Cephelaxin |
| 1337 | Cat | R | R | R | I | R | I | I | I | R |
| 1626 | Cat | R | R | R | I | R | I | I | S | R |
| 1917 | Cat | R | R | R | R | R | R | R | R | R |
| 1954 | Dog | R | R | R | R | R | R | R | R | R |
| 1994 | Dog | R | R | S | S | S | S | S | S | S |
| 2157 | Dog | R | R | R | R | R | S | R | R | R |
| 2165 | Dog | R | R | R | R | R | R | R | I | R |
| 2191 | Cat | R | R | R | R | R | R | R | R | R |
| 2233 | Cat | R | S | I | I | I | S | S | S | S |
| 2265 | Dog | R | R | R | R | R | R | R | R | R |
| 2277 | Dog | R | R | R | R | R | S | S | S | R |
| 2294 | Dog | R | R | R | R | R | R | R | R | R |
| 2517 | Dog | R | R | I | R | I | S | S | S | I |
| 2544 | Cat | R | R | R | R | R | R | R | R | R |
| 2551 | Dog | R | R | R | R | R | R | I | S | R |
| 2555 | Dog | R | R | R | R | R | R | R | R | R |
| 2561 | Cat | R | R | R | R | R | R | R | R | R |
| 2591 | Dog | R | R | S | I | S | S | S | S | I |
| 2648 | Dog | R | R | R | R | R | S | I | S | R |
| 2674 | Dog | R | R | R | R | R | R | R | I | R |
| 2697 | Dog | R | R | R | R | R | R | R | I | R |
| 2702 | Dog | R | R | R | R | R | R | R | I | R |
| 2715 | Cat | R | R | R | R | R | R | I | S | R |
| 2725 | Dog | R | R | R | R | R | R | I | R | R |
| 2734 | Dog | R | R | R | R | R | R | I | I | R |
| 2750 | Dog | R | R | R | R | R | R | R | R | R |
| 2755 | Dog | R | R | R | R | R | R | R | R | R |
| 2768 | Dog | R | R | R | R | R | S | R | S | R |
| 2777 | Dog | R | R | R | R | R | R | R | R | R |
| 2812 | Dog | R | R | R | R | R | I | I | I | R |
| 2813 | Dog | R | R | R | R | R | I | R | I | R |
| 2814 | Dog | R | R | R | R | R | R | R | I | R |
| 2815 | Cat | R | R | S | R | S | S | S | S | S |
| 2830 | Dog | R | R | R | R | R | I | R | S | R |
| 2837 | Dog | R | R | R | R | R | R | S | R | R |
| 2844 | Dog | R | R | R | R | R | S | R | S | R |
| 2851 | Cat | R | R | R | R | R | R | R | R | R |
| 2855 | Dog | R | R | S | I | I | I | S | I | I |
| 2868 | Dog | R | R | S | S | S | S | S | S | S |
| 2872 | Cat | R | R | R | R | R | I | I | R | R |
| 2877 | Dog | R | R | R | R | R | R | R | R | R |
| 2880 | Dog | R | R | R | R | R | R | R | R | R |
| 2899 | Dog | R | R | R | R | R | R | R | R | R |
| 2900 | Dog | R | R | R | R | R | R | R | R | R |
| 2901 | Dog | R | R | R | I | R | I | S | S | R |
| 2903 | Cat | R | R | R | R | R | R | I | R | R |
| 2904 | Cat | R | R | R | R | R | R | I | R | R |
| 2915 | Dog | R | R | R | R | R | S | I | S | R |
| 2930 | Dog | R | R | I | S | S | S | S | S | I |
| 2936 | Dog | R | R | I | S | S | S | S | S | S |
| 2937 | Cat | R | R | R | R | R | R | R | R | R |
| 2938 | Cat | R | R | R | R | R | R | R | R | R |
| 2947 | Dog | R | R | I | I | I | S | S | S | I |
| 2953 | Dog | R | R | R | R | R | R | R | R | R |
| 2956 | Dog | R | R | R | R | R | R | R | R | R |
| 2957 | Dog | R | R | I | I | I | S | S | S | I |
| 2962 | Cat | R | R | R | R | R | R | R | R | R |

S: susceptible, I: intermediate, R: resistant

Table 3 Antimicrobial susceptibility testing of ESBL and/or pAmpC-producing *K. pneumoniae* (con.)

| Isolate  number | Species | Antimicrobials | | | | | | | | | |
| --- | --- | --- | --- | --- | --- | --- | --- | --- | --- | --- | --- |
|  |  | Cephalothin | Ciprofloxacin | Doxycycline | Enrofloxacin | Gentamycin | Imipenem | Neomycin | Cefoxitin | SXT | Tetracycline |
| 1337 | Cat | R | R | R | R | S | S | R | R | S | R |
| 1626 | Cat | R | R | S | R | S | S | I | R | S | S |
| 1917 | Cat | R | R | R | R | S | S | I | R | S | R |
| 1954 | Dog | R | R | R | R | R | S | R | R | R | R |
| 1994 | Dog | S | I | R | S | S | S | S | S | R | R |
| 2157 | Dog | R | R | R | R | R | S | R | R | R | R |
| 2165 | Dog | R | R | R | R | R | S | R | R | R | R |
| 2191 | Cat | R | R | R | R | S | S | R | R | R | R |
| 2233 | Cat | S | S | S | S | S | S | I | S | S | S |
| 2265 | Dog | R | R | R | R | R | S | R | R | R | R |
| 2277 | Dog | R | R | R | R | R | S | I | R | R | R |
| 2294 | Dog | R | R | I | R | R | S | R | R | R | S |
| 2517 | Dog | I | I | I | I | S | S | S | S | S | I |
| 2544 | Cat | R | R | R | R | R | I | R | R | R | R |
| 2551 | Dog | R | S | R | I | R | S | R | R | R | R |
| 2555 | Dog | R | R | R | R | R | S | I | R | S | R |
| 2561 | Cat | R | R | R | R | R | I | R | R | R | R |
| 2591 | Dog | I | S | S | S | S | S | I | S | S | S |
| 2648 | Dog | R | I | R | I | S | S | I | I | S | R |
| 2674 | Dog | R | R | R | R | R | S | R | R | R | R |
| 2697 | Dog | R | R | R | R | R | I | R | R | R | I |
| 2702 | Dog | R | R | R | R | I | S | R | R | R | S |
| 2715 | Cat | R | I | R | I | S | S | I | R | R | R |
| 2725 | Dog | R | I | R | R | R | S | R | R | R | R |
| 2734 | Dog | R | S | R | R | I | S | R | R | R | R |
| 2750 | Dog | R | R | I | R | R | S | R | R | R | R |
| 2755 | Dog | R | R | R | R | R | I | R | R | S | R |
| 2768 | Dog | R | S | R | I | R | S | R | R | R | R |
| 2777 | Dog | R | R | R | R | R | S | R | R | R | R |
| 2812 | Dog | R | S | R | R | R | I | I | R | R | R |
| 2813 | Dog | R | S | R | R | R | I | R | R | S | R |
| 2814 | Dog | R | S | R | R | R | I | R | R | R | R |
| 2815 | Cat | R | I | S | I | S | S | S | S | S | S |
| 2830 | Dog | R | R | R | R | R | S | R | R | R | R |
| 2837 | Dog | R | R | R | R | R | S | I | R | R | R |
| 2844 | Dog | R | R | R | R | R | S | I | S | R | R |
| 2851 | Cat | R | R | R | R | S | S | I | S | S | I |
| 2855 | Dog | I | S | R | S | S | S | I | R | S | S |
| 2868 | Dog | S | R | R | R | S | S | S | S | R | R |
| 2872 | Cat | R | R | R | R | S | S | S | S | S | R |
| 2877 | Dog | R | R | R | R | R | S | I | R | R | R |
| 2880 | Dog | R | R | S | R | R | S | S | R | S | S |
| 2899 | Dog | R | R | R | R | R | R | I | R | R | R |
| 2900 | Dog | R | R | R | R | R | R | R | S | R | R |
| 2901 | Dog | R | S | R | I | R | S | S | R | R | R |
| 2903 | Cat | R | R | R | R | S | I | I | R | S | I |
| 2904 | Cat | R | R | I | R | S | I | S | R | S | S |
| 2915 | Dog | R | R | R | R | R | S | S | R | R | R |
| 2930 | Dog | I | S | I | S | S | S | S | S | S | S |
| 2936 | Dog | I | S | S | S | S | S | S | S | S | S |
| 2937 | Cat | R | R | I | R | S | R | I | R | S | S |
| 2938 | Cat | R | R | I | R | S | S | I | R | S | S |
| 2947 | Dog | I | S | R | I | S | S | I | S | S | S |
| 2953 | Dog | R | R | R | R | R | S | I | R | R | R |
| 2956 | Dog | R | R | R | R | I | R | R | R | S | R |
| 2957 | Dog | I | S | R | I | S | S | I | S | S | R |
| 2962 | Cat | R | R | R | R | I | S | R | R | R | R |

S: susceptible, I: intermediate, R: resistant
